# Prehospital Intubation and Outcome in Traumatic Brain Injury—Assessing Intervention Efficacy in a Modern Trauma Cohort

**DOI:** 10.3389/fneur.2018.00194

**Published:** 2018-04-10

**Authors:** Rebecka Rubenson Wahlin, David W. Nelson, Bo-Michael Bellander, Mikael Svensson, Adel Helmy, Eric Peter Thelin

**Affiliations:** ^1^Department of Clinical Science and Education, Södersjukhuset, Karolinska Institutet, Stockholm, Sweden; ^2^Department of Anesthesia and Intensive Care, Södersjukhuset, Stockholm, Sweden; ^3^Section of Anesthesiology and Intensive Care, Department of Physiology and Pharmacology, Karolinska Institutet, Stockholm, Sweden; ^4^Department of Clinical Neuroscience, Karolinska Institutet, Stockholm, Sweden; ^5^Department of Neurosurgery, Karolinska University Hospital Solna, Stockholm, Sweden; ^6^Division of Neurosurgery, Department of Clinical Neurosciences, University of Cambridge, Cambridge, United Kingdom

**Keywords:** traumatic brain injury, advanced airway management, prehospital trauma care, human, emergency medical services

## Abstract

**Background:**

Prehospital intubation in traumatic brain injury (TBI) focuses on limiting the effects of secondary insults such as hypoxia, but no indisputable evidence has been presented that it is beneficial for outcome. The aim of this study was to explore the characteristics of patients who undergo prehospital intubation and, in turn, if these parameters affect outcome.

**Material and methods:**

Patients ≥15 years admitted to the Department of Neurosurgery, Stockholm, Sweden with TBI from 2008 through 2014 were included. Data were extracted from prehospital and hospital charts, including prospectively collected Glasgow Outcome Score (GOS) after 12 months. Univariate and multivariable logistic regression models were employed to examine parameters independently correlated to prehospital intubation and outcome.

**Results:**

A total of 458 patients were included (*n* = 178 unconscious, among them, *n* = 61 intubated). Multivariable analyses indicated that high energy trauma, prehospital hypotension, pupil unresponsiveness, mode of transportation, and distance to the hospital were independently correlated with intubation, and among them, only pupil responsiveness was independently associated with outcome. Prehospital intubation did not add independent information in a step-up model versus GOS (*p* = 0.154). Prehospital reports revealed that hypoxia was not the primary cause of prehospital intubation, and that the procedure did not improve oxygen saturation during transport, while an increasing distance from the hospital increased the intubation frequency.

**Conclusion:**

In this modern trauma cohort, prehospital intubation was not independently associated with outcome; however, hypoxia was not a common reason for prehospital intubation. Prospective trials to assess efficacy of prehospital airway intubation will be difficult due to logistical and ethical considerations.

## Introduction

Traumatic brain injury (TBI) constitutes a major public health issue every year for approximately 10 million people globally (1). Prehospital TBI management focuses on prevention of secondary insults, such as prehospital hypoxia (blood oxygen saturation <90%) and hypotension [systolic blood pressure (SBP) <90 mmHg], which have been shown to lead to intracranial lesion deterioration as well as unfavorable long-term outcome (2–6). Current regional guidelines state that a compromised airway should be secured in TBI patients, especially when a long prehospital transport time is expected, or when hypoxia cannot be corrected by other means (7). Consequently, endotracheal intubation is recommended for TBI patients with a prehospital Glasgow Coma Scale (GCS) ≤8 (unconscious), as is suggested by the Brain Trauma Foundation (7, 8). Unconscious patients may lose protective airway reflexes which may lead to aspiration (9), as well as to obstruction of a collapsed epiglottis, tongue, and soft palate, conditions leading to hypoxia (10). By providing immediate care at the trauma scene, ensuring appropriate airway management, oxygenation, and adequate blood pressure, improvement in outcome has been shown (8, 11, 12). However, due to its complexity, prehospital intubation in TBI patients is a procedure that can itself result in hypoxia (13, 14), hypotension (15), or even hypertension (16, 17), complications especially unfavorable for TBI patients. It has also been established that when performed poorly, the procedure is hazardous and might even worsen outcome (18–21). Moreover, two other factors shown to influence outcome in trauma is the prehospital duration (“the golden hour”) (22) and the distance to the hospital (23), of course both closely related. Although a large number of studies on prehospital intubation have been conducted, there are only a few on the relationship between advanced prehospital airway management and the distance to hospital. Generally, those studies that have addressed the correlation between prehospital time duration and intubation have not uniquely focused on TBI patients (24–27).

In 2008, the Scandinavian guidelines for prehospital management of severe TBI were published to guide and standardize prehospital care (7) and were also implemented regionally. These guidelines stressed the need for standardized prehospital treatment for patient suffering from suspected TBI. Today, there is no clear consensus on whether prehospital intubation improves outcome, supported by a meta-analysis (28). Some main reasons for this are the lack of good prospective trials and that retrospective trials have difficulties adjusting for the treatment and selection bias. While this study does not constitute a prospective trial, it aims to provide detailed information from a modern prehospital trauma care system containing detailed information from hospital charts and prospectively gathered outcome data.

In contrast to similar studies, we wished to primarily analyze the characteristics of patients who underwent prehospital intubation, and in turn, which of these factors that independently affected long-term functional outcome. As a secondary aim, we analyzed different aspects of the prehospital management logistics, focusing on the role of prehospital intubation.

## Materials and Methods

### Ethics and Study Design

The study received ethical approval from the Regional Ethical Review Board in Stockholm reference numbers 2007/1113-31, 2010/1979-32, 2013/1718-32, 2014/691-32, and 2015/1675-31/1. This is an observational cohort study of TBI patients.

### Study Population

Included patients were; adult and late adolescent trauma patients (≥15 years of age) with prehospital trauma charts, a computer tomography verified TBI (ICD-10 S06.2-S06.9) treated at the only neurosurgical unit (at Karolinska University Hospital, Stockholm, Sweden) in the region during the period January first 2008 to December 31st 2014 in Stockholm, Sweden (following prehospital guideline implementation). Patients were excluded if declared dead on scene, admitted to the reporting hospital >6 h after the trauma or in cases when the exact time of trauma was unknown. In addition, we excluded patients transported from another county for specialist care and/or transfers after >24 h to the university hospital after admission to any of the other hospitals.

### Prehospital Data Collection

Data were collected from the neuro trauma registry at the Karolinska University Hospital. Prehospital data were retrieved from the electronic prehospital records network (CAK-net) used by all ambulance caregivers. The ambulances are equipped with a global position satellite system (GPS) that delivers a GPS coordinate according to the SWEREF 99 (Swedish reference frame 1999) system (29). The SWEREF 99 has been shown to have a margin of error within 0.5 m of the WGS 84 (World Geodetic System 1984) that the commercially available GPS system uses as reference (29). The electronic prehospital records also provide the exact address on the scene of accident. If the SWEREF 99 coordinates were not available, Google Maps^®^ was used to generate the WGS 84 coordinates using the entered address (used for *n* = 161, 35%). The preferred ambulance route from the scene of accident to the primary hospital was chosen. Travel distances were adjusted for recent infrastructure projects in the Stockholm region during the study period to indicate the correct paths for the ambulances. The first author (Rebecka Rubenson Wahlin) who is an experienced staff member of the Stockholm Emergency Medical Services (EMS) did perform these assessments. For helicopter transport, the linear distance to the hospital was used.

### Clinical Variables

Age and gender were included from hospital charts. Mechanism of injury was included from prehospital records. Multitrauma, defined as an injury to any other major organ system except the head and spine, were noted (30). The energy of the trauma, as defined by advanced trauma and life-support guidelines (31), were defined as “low energy” or “high energy,” if available. Prehospital hypoxia was defined as a peripheral oxygen saturation <90%, and a prehospital hypotension if the SBP <90 mmHg, at any time during the prehospital duration. If serum ethanol was positive at admittance to the hospital, it was noted as it has been shown to be associated with a favorable outcome (32). GCS was noted, and “unconscious” patients were defined as a GCS ≤ 8 at the scene of accident (33). If one, or two, pupil(s) presented without light reflex, it was defined as “pupil unresponsiveness.” To assess the neuro-radiological damage, we assessed the admission CT scans according to Marshall (34) classification, Rotterdam CT-score (35), and Stockholm CT-scores (36). We chose to use the Stockholm CT-scores in the analysis as they are presented as continuous variables where higher levels and have been shown to best correlate to outcome (36). Moreover, head abbreviated injury scale (AIS) > 3, as defined as at least a “severe” TBI, were noted together with injury severity score (ISS) and new injury severity score (NISS) (37). S100B, a protein of brain tissue fate and a potent biomarker of brain injury (38), were assessed at admission and at 12–48 h after injury as later samples have been shown to be less influenced by extracranial trauma (39, 40). Intensive care unit stay was defined as the length of stay in days. Survival status was noted, as well as 12 months Glasgow Outcome Score (GOS) (33) assessed by clinic visits and questionnaires regarding health-related quality of life.

The prehospital variables were collected and defined in accordance with The Utstein Trauma Template (41) and Utstein-style template for prehospital airway management (42) to increase the possibility to compare data with other prehospital studies; time from alarm until hospital arrival, highest level of prehospital care provided, prehospital airway management, type of prehospital airway management, and type of transportation, time from alarm until arrival at scene were all extracted from the prehospital records as well as SBP, respiratory rate, heart rate and GCS on scene, indication for airway intervention, attempts of airway intervention, intubation success, device used in success, and post intervention ventilation.

The time periods were defined as follows; time on scene and the time of departure from scene until hospital arrival were defined in minutes and seconds, the distance from scene of accident to hospital were defined in kilometers.

The saturation from pulse oximetry devices were acquired from the scene and at arrival at the hospital, this “delta-saturation” (oxygen saturation at the emergency department—oxygen saturation at the scene) was reported.

### Prehospital Conditions

The Stockholm County Council (SCC) includes 26 municipalities covering 6,519 square kilometers, an archipelago of approximately 30,000 islands, and is responsible for the EMS of 2.1 million inhabitants (43). The SCC responsibility includes both the EMS and the seven emergency hospitals, of which, solely one is a level-1 trauma center according to the American College of Surgeons’ criteria (44). The EMS are provided by one SCC owned company and by two private companies contracted by the SCC. One Emergency Medical Communications Centre operates in the area.

During the study period (2008–2014), there were 55–61 ground ambulances, and three rapid-response vehicles during daytime (07:00–20:00) (43). A rapid-response vehicle was physician-manned and the two others by nurse anesthetists, as well as emergency medical technicians (EMTs). All ground-based ambulances were manned by two people, an EMT and one registered nurse. During nighttime, there is no physician on call, and about 38 ambulances operate in the area (45). In addition, there is also a nurse anesthetist manned helicopter (one additional helicopter during summer time) and one mobile intensive care unit operating in the area.

As per the new guidelines that were implemented in 2008, registered nurses may administer drugs and handle the laryngeal mask after personal delegation (46). Nurse anesthetists with more than 1 year of clinical experience are also allowed to perform prehospital endotracheal intubation (PHETI) without drugs (46). Nurse anesthetists with more than 3 years of experience may perform drug-assisted rapid sequence induction after personal delegation.

### Statistical Analysis

For descriptive purposes, continuous data are presented as medians with interquartile ranges (except the normally distributed variable age as mean and SD). Mann–Whitney *U*-test and Chi-square test were used to compare continuous and categorical parameters, respectively. A univariate regression analysis was used to correlate factors to prehospital intubation (“lrm” function in R, “rms”-package) (47). For outcome prediction, a similar univariate proportional odds regression was used toward GOS levels. We know from previous studies using the same database that the proportional odds of GOS levels results in similar results as dichotomizing it into GOS levels 1–3 versus 4–5 (38, 40). In the two univariate models, un-imputed data were used. Nagelkerke’s pseudo-*R*^2^ was used to illustrate the pseudo explained variance, where “0” does not provide any variance while “1” fully explains the model. Multivariable models, utilizing Multiple Imputation (MI) (“mice”-package in R), including all parameters significant in the univariate analyses, were performed to determine factors independently correlated to intubation and functional outcome. Only parameters significant in univariate analyses were included in the multivariate models and the models were bias-adjusted for multiple parameters. Dependant variables were GOS or prehospital intubation. To examine how prehospital intubation affected outcome in the multivariate model, a step-up procedure where used. Conditional density plots and box plots were used to illustrate continuous versus categorical variables and box plots comparing continuous variables (delta-saturation).

The statistical program R was used, utilizing the interface R-studio Version 0.99.902 (47). The statistical significance level was set to *p* < 0.05.

### Missing Data

Some data were missing from the hospital charts and were imputed in order to optimize multivariate analyses, thus being able to utilize all patients. MIs (“mice” package in R) were performed, retaining seven imputed dataset, which were used to look for parameters independently correlated to functional outcome and prehospital intubation. The current method is recommended in this type of multivariate analyses, as is advocated by the statistical literature as well as the IMPACT research group (48, 49).

## Results

### Patient Demographics

During the period January first 2008 to December 31st 2014, 738 TBI patients were considered for inclusion and, out of these, 122 patients were excluded due to missing prehospital records, 75 patients due to uncertain trauma time or admittance more than 6 h after trauma, and 83 patients as they had been referred from other counties (i.e., secondary transports). In total, 458 patients fulfilled inclusion criteria. Demographics for all patients, as well as missing data for each parameter, are presented (Table S1 in Supplementary Material). Out of these 458 patients, 178 were unconscious at the scene of accident and thus represented patients in potential need of prehospital airway management according to the implemented guidelines. Among the 178 unconscious patients, 61 were intubated (a total of 66 were intubated, but in five cases, this was because the patient was conscious, but uncooperative or combative at the scene).

The unconscious group was more severely injured (according to all classifications), with higher in-hospital mortality and worse long-term functional outcome compared to the conscious patients (Table 1). In the unconscious cohort, the intubated patients were almost 10 years younger (38.8 versus 48.9 years), more often victims of high-energy trauma (however, this parameter must be interpreted with caution due to the amount of missing data) and were more often transported by helicopter (52% compared to 16% for non-intubated patients) (Table 2). The intubated group also had a longer distance from scene of accident to the hospital (in median almost 10 km to the hospital) and were longer at-scene as compared to the non-intubated patients. The intubated patients remained in median 12 min longer at the scene of accident (Table 2).

**Table 1:** Patient characteristics and outcome data between conscious and unconscious patients.

	Conscious (*n* = 280)	Unconscious (*n* = 178)	*p*-Value
**Prehospital data**			
Age, years (SD)	48.9 (19.6)	45.3 (19.2)	0.055
Gender, male (%)	*n* = 204 (73%)	*n* = 131 (74%)	0.948
Multitrauma, *n* (%)	*n* = 52 (19%)	*n* = 79 (44%)	<0.001
Positive blood ethanol, *n* (%)	*n* = 100 (36%) (24 missing, 9%)	*n* = 75 (42%) (7 missing, 4%)	0.375
Hypoxia at SoA	*n* = 9 (3%) (20 missing, 7%)	*n* = 32 (18%) (17 missing, 10%)	<0.001
Hypotension at SoA	*n* = 6 (2%) (23 missing, 8%)	*n* = 9 (5%) (34 missing, 19%)	0.088
Trauma energy, high *n* (%)	*n* = 34 (12%) (172 missing, 61%)	*n* = 68 (38%) (78 missing, 44%)	<0.001

**Hospital data**			
Pupil unresponsiveness, *n* (%)	*n* = 19 (7%) (6 missing, 2%)	*n* = 61 (34%) (6 missing, 3%)	<0.001
Stockholm CT Score	1.9 (1–2.5)	3 (2–3.5)	<0.001
Head-AIS > 3	*n* = 191 (68%)	*n* = 157 (88%)	<0.001
ISS, median IQR	21.5 (13–26)	26 (22–38)	<0.001
NISS, median IQR	29 (24–41)	48 (34–57)	<0.001
S100B admission, median μg/L	0.99 (0.36–2.35) (68% missing)	2.9 (1.4–7.35) (34% missing)	<0.001
S100B peak at 12–48 h, median μg/L	0.22 (0.13–0.42) (38% missing)	0.36 (0.20–0.74) (8% missing)	<0.001
Hospital length of stay (LOS), median days (IQR)	9 (5–19)	20 (9–34)	<0.001
ICU LOS, median days (IQR)	1.7 (0–7)	10.6 (3–19)	<0.001

**Outcome data**			
In-hospital mortality	*n* = 19 (7%)	*n* = 31 (17%)	0.001
Long-term GOS 1–3 (unfavorable), *n* (%)	*n* = 81 (29%)	*n* = 96 (54%)	<0.001

*Table illustrating the demographic data between conscious and unconscious patients. Missing data are mentioned for each parameter, if present. Difference between groups are compared using chi-square or Mann–Whitney test, were applicable*.

*SoA, scene of accident; CT, computerized tomography; AIS, Abbreviated Injury Scale; ISS, Injury Severity Score; NISS, New Injury Severity Score; ICU, intensive care unit; GOS, Glasgow Outcome Score; IQR, interquartile range*.

**Table 2:** Patient characteristics and outcome data, intubated and non-intubated groups among unconscious patients.

Parameters	Not intubated (*n* = 117)	Intubated (*n* = 61)	*p*-Value
**Prehospital data**			
Age, years (SD)	48.9 (18.7)	38.8 (18.7)	0.001
Gender, male (%)	*n* = 86 (74%)	*n* = 43 (73%)	0.618
Multitrauma, *n* (%)	*n* = 42 (36%)	*n* = 37 (61%)	0.003
Positive blood ethanol, *n* (%)	*n* = 54 (46%) (missing 6%)	*n* = 21 (34%)	0.091
Hypoxia at SoA	*n* = 19 (16%) (missing 12%)	*n* = 13 (22%)	0.689
Hypotension at SoA	*n* = 2 (20% missing)	*n* = 7 (11%)	0.015
Trauma energy, high, *n* (%)	*n* = 26 (22%) (missing 58%)	*n* = 42 (69%) (missing 16%)	0.003

**Hospital data**			
Pupil unresponsiveness, *n* (%)	*n* = 30 (27%) (missing 4%)	*n* = 31 (51%) (missing 1%)	0.002
Stockholm CT Score	2.9 (2.0–3.5)	3.0 (2.0–3.5)	0.877
Head-AIS > 3	*n* = 106 (91%)	*n* = 53 (87%)	0.641
ISS, median IQR	26 (21–34)	29 (25–42)	0.005
NISS, median IQR	43 (34–57)	50 (34–57)	0.237
S100B admission, median μg/L	2.7 (1.3–4.9) (missing 41%)	4.6 (1.7–11) (missing *n* = 14, 23%)	0.093
S100B peak at 12–48 h, median μg/L	0.38 (0.21–0.80) (missing 9%)	0.33 (0.20–0.69) (missing 8%)	0.456
Hospital length of stay (LOS), median days (IQR)	19 (9–33)	22 (8–35)	0.700
ICU LOS, median days (IQR)	10 (3–18)	13 (4–22)	0.194

**Outcome data**			
In-hospital mortality	*n* = 21 (18%)	*n* = 10 (16%)	0.959
Long-term GOS 1–3 (unfavorable), *n* (%)	*n* = 57 (49%)	*n* = 39 (64%)	0.076

**Prehospital transportation data**			
Transported with helicopter, *n* (%)	*n* = 19 (16%)	*n* = 32 (52%)	<0.001
Time from alarm until hospital arrival, mm:ss, median (IQR)	36:29 (28:04–47:57)	49:34 (37:33–60:08)	<0.001
Time from alarm until arrival at scene, mm:ss, median (IQR)	09:38 (06:51–14:49)	12:56 (09:00–20:50)	0.013
On-scene time, mm:ss, median (IQR)	14:31 (10:23–21:14)	26:40 (21:01–16:41)	<0.001
Time from scene until hospital arrival, mm:ss, median (IQR)	10:07 (06:29–15:24)	10:35 (06:27–16:59)	0.731
Distance from scene of accident to the hospital, median kilometers (IQR)	9.2 (5.1–18.1)	17.2 (10.8–32.22)	<0.001

*Table illustrating the demographic data between intubated and non-intubated patients. Missing data are mentioned for each parameter, if present. Difference between groups are compared using chi-square or Mann–Whitney test, were applicable*.

*SoA, scene of accident; CT, computerized tomography; AIS, Abbreviated Injury Scale; ISS, Injury Severity Score; NISS, New Injury Severity Score; ICU, intensive care unit; GOS, Glasgow Outcome Score; IQR, interquartile range; mm, minutes; ss, seconds*.

### Parameters Correlated to Prehospital Intubation

The parameters that were independently associated with prehospital intubation among the unconscious patients were mode of transportation (by helicopter), amount of energy involved in the trauma, time from alarm to hospital arrival, pupil responsiveness, prehospital hypotension, and distance from trauma scene to the hospital (Table 3). A multiregression toward prehospital intubation using significant variables in the univariate regression exhibited an adjusted pseudo-*R*^2^ of 0.393 (Table 3). Notably, prehospital hypoxia was not significantly correlated to prehospital intubation in univariate analysis for the unconscious patients (*p* = 0.547).

**Table 3:** Parameters correlated with prehospital intubation in the unconscious population.

Univariate analysis	For unconscious patients (*n* = 178)		
Parameter (s)	*p*-Value	Pseudo-*R*^2^	Correlation coefficient
Age	<0.001	0.085	–
Gender	0.500	NS	NS
Multitrauma	0.002	0.075	+
High/low energy trauma*	<0.001	0.160	+
Positive blood ethanol	0.063	NS	NS
Prehospital hypoxia	0.547	NS	NS
Prehospital hypotension*	0.006	0.070	+
Pupil responsiveness*	<0.001	0.095	+
Stockholm CT Score	0.816	NS	NS
AIS	0.475	NS	NS
ISS	<0.001	0.086	+
NISS	0.141	NS	NS
S100B admission	0.031	0.053	+
S100B 12–48 h	0.549	NS	NS
Mode of transportation (helicopter)*	<0.001	0.181	+
Distance from trauma to hospital	0.003	0.068	+
Time from alarm to hospital arrival	<0.001	0.121	+
Time from alarm until EMS arrival at scene	0.022	0.041	+
EMS on-scene time*	<0.001	0.165	+
Time from scene to hospital arrival	0.328	NS	NS

**Multivariable analysis**		**Adjusted pseudo-*R*^2^**	

*Parameters independently correlated with prehospital intubation.	<0.001	0.393	

*Parameters significant in a bivariate regression analysis versus prehospital intubation with a un-imputated dataset, p-value for significance, Nagelkerke’s pseudo-R^2^ for the explained variance and correlation coefficient if an increase of the parameters was positively or negatively correlated to pre-hospital intubation. In the multivariate proportional odds model, an imputated dataset was used. Due to co-variance of the parameters, time for EMS on scene was the only time duration that was used*.

*NS, not significant; CT, computerized tomography; AIS, Abbreviated Injury Scale; ISS, Injury Severity Score; NISS, New Injury Severity Score; EMS, Emergency medical services*.

Predictably, if all 458 patients were included in the model (Table S2 in Supplementary Material), the parameter “Unconscious” had the strongest association toward prehospital intubation (pseudo-*R*^2^ 0.361). Apart from that, the combined patient cohort presented similar results (Table S2 in Supplementary Material).

### Parameters Correlated to Long-Term Functional Outcome

The parameters that independently correlated to functional outcome in the multivariate proportional odds analysis of unconscious patients were: levels of the biomarker S100B 12–48 h after trauma, Stockholm CT-score, NISS, age, and pupil responsiveness (Table 4). This model exhibited an adjusted pseudo explained variance in relation to long-term GOS of 0.502 (we defined this as our “base” model). Prehospital intubation did not significantly correlate to outcome in univariate analysis (*p* = 0.296), and did not add any significant independent information to the base model (*p* = 0.154) (Table 4). In an exploratory approach, we analyzed the unconscious patients who had prehospital hypoxia (*n* = 32) to see if intubation specifically improved outcome in this cohort, but could not see any significant association (*p* = 1.0, data not shown).

**Table 4:** Parameters correlated to functional outcome in the unconscious cohort.

Univariate analysis	For unconscious patients (*n* = 178)		
Parameter	*p*-Value	Pseudo-*R*^2^	Correlation coefficient
Age*	<0.001	0.089	–
Gender	0.219	NS	NS
Multitrauma	0.475	NS	NS
High/low energy trauma	0.170	NS	NS
Positive blood ethanol level	<0.001	0.074	+
Prehospital hypoxia	0.016	0.037	–
Prehospital hypotension	0.142	NS	NS
Prehospital intubation	0.296	NS	NS
Pupil responsiveness*	<0.001	0.082	–
Stockholm CT score*	<0.001	0.164	–
AIS	0.026	0.030	–
ISS	<0.001	0.069	–
NISS*	<0.001	0.099	–
S100B admission	<0.001	0.153	–
S100B 12–48 h*	<0.001	0.302	–
Distance from trauma to hospital	0.496	NS	NS
Mode of transportation	0.212	NS	NS
Time from alarm to hospital arrival	0.867	NS	NS
Time for EMS to reach the trauma scene	0.423	NS	NS
Time for EMS on scene	0.692	NS	NS
Time from scene to hospital arrival	0.512	NS	NS

Multivariable analysis		**Adjusted pseudo-*R*^2^**	

*Parameters independently correlated to outcome	<0.001	0.502	
Significant parameters + prehospital intubation	0.154	0.504	

*Parameters significant in a proportional odds regression analysis versus outcome (GOS1-5) with an un-imputated dataset, *p*-value for significance, Nagelkerke’s pseudo-*R*^2^ for the explained variance and correlation coefficient if an increase of the parameters was positively or negatively correlated to an increase in GOS (better outcome). In the multivariate proportional odds model, an imputated dataset was used. Due to co-variance of the parameters, S100B 12–48 h was preferred to admission S100B, and NISS was preferred to AIS and ISS in the model. A step-up model was used to see if prehospital intubation added independent information to the multivariate model*.

*NS, not significant; CT, computerized tomography; AIS, abbreviated injury scale; ISS, injury severity score; NISS, New Injury Severity Score; EMS, Emergency medical services*.

When assessing the combined patient cohort of 458 patients, similar correlations toward outcome were found, with the obvious addition of “unconscious” patients having a more unfavorable outcome (Table S3 in Supplementary Material). Prehospital hypoxia was an independent predictor of unfavorable outcome in the combined cohort, as well as prehospital intubation (Table S3 in Supplementary Material). Interestingly, neither “distance from the trauma to the hospital” nor the “total prehospital” or “on-scene” times were correlated to the long-term outcome (Table 3; Table S3 in Supplementary Material).

### Logistics of Prehospital Airway Management

Of the 178 unconscious patients, 61 patients (41%) were in need of PHETI for different reasons, a majority were intubated due to decreased level of consciousness (40%) or “ineffective ventilation” (18%), only two (3%) were intubated primarily due to hypoxia according to the prehospital charts (Table 5). Out of the patients who were *conscious* at the scene of accident, *n* = 5 were intubated. In none of these cases was the airway compromised, instead, these patients were sedated due to psychomotor agitation (Table 5). The number of intubation attempts varied, but in 85% of the cases, only one intubation attempt was necessary (Table S1 in Supplementary Material). There were nine failed intubations at the scene of accident. In an exploratory sub-group analysis, long-term GOS were neither related to multiple intubation attempts, nor failed intubation in the unconscious cohort (data not shown).

**Table 5:** Reason for prehospital endotracheal intubation.

Reason for endotracheal intubation	*n* = 66 (% of intubated patients)
1. Decreased level of consciousness	*n* = 26 (40%)
2. Hypoxemia	*n* = 2 (3%)
3. Ineffective ventilation	*n* = 12 (18%)
4. Existing airway obstruction	*n* = 8 (12%)
5. Impending airway obstruction	*n* = 9 (14%)
6. Combative or uncooperative	*n* = 5 (8%)
7. Relief or pain or distress	*n* = 1 (2%)
8. Cardiopulmonary arrest	*n* = 3 (5%)

*Primary reason for endotracheal intubation, as stated in prehospital trauma charts*.

Moreover, we could not detect any difference in the intubation success rate depending on care provider, EMS physician, or nurse (*p* = 0.423, data not shown).

**Figure 1:**
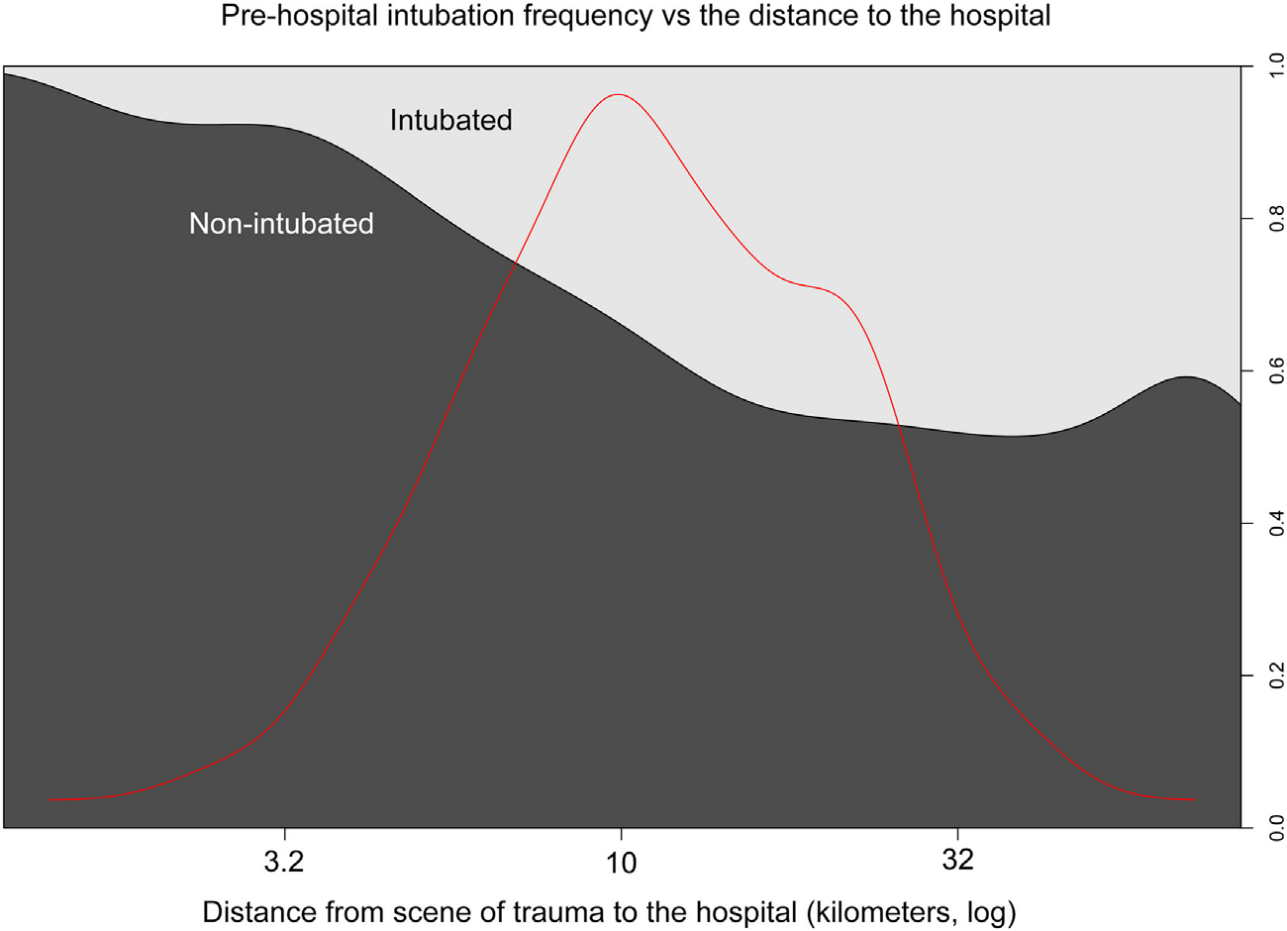
Intubation frequency and prehospital transport distance. Distance from the trauma to the hospital (*x*-axis, kilometers log) and the proportion of prehospital intubation (*y*-axis right). Bright represents intubated- and dark non-intubated patients (*y*-axis left). The red line represents the data distribution.

With increasing distance from the scene of accident, the rate of prehospital intubation escalated and at >10 km almost 50% of all patients were intubated (Figure 1), in line with the introduced guidelines.

**Figure 2:**
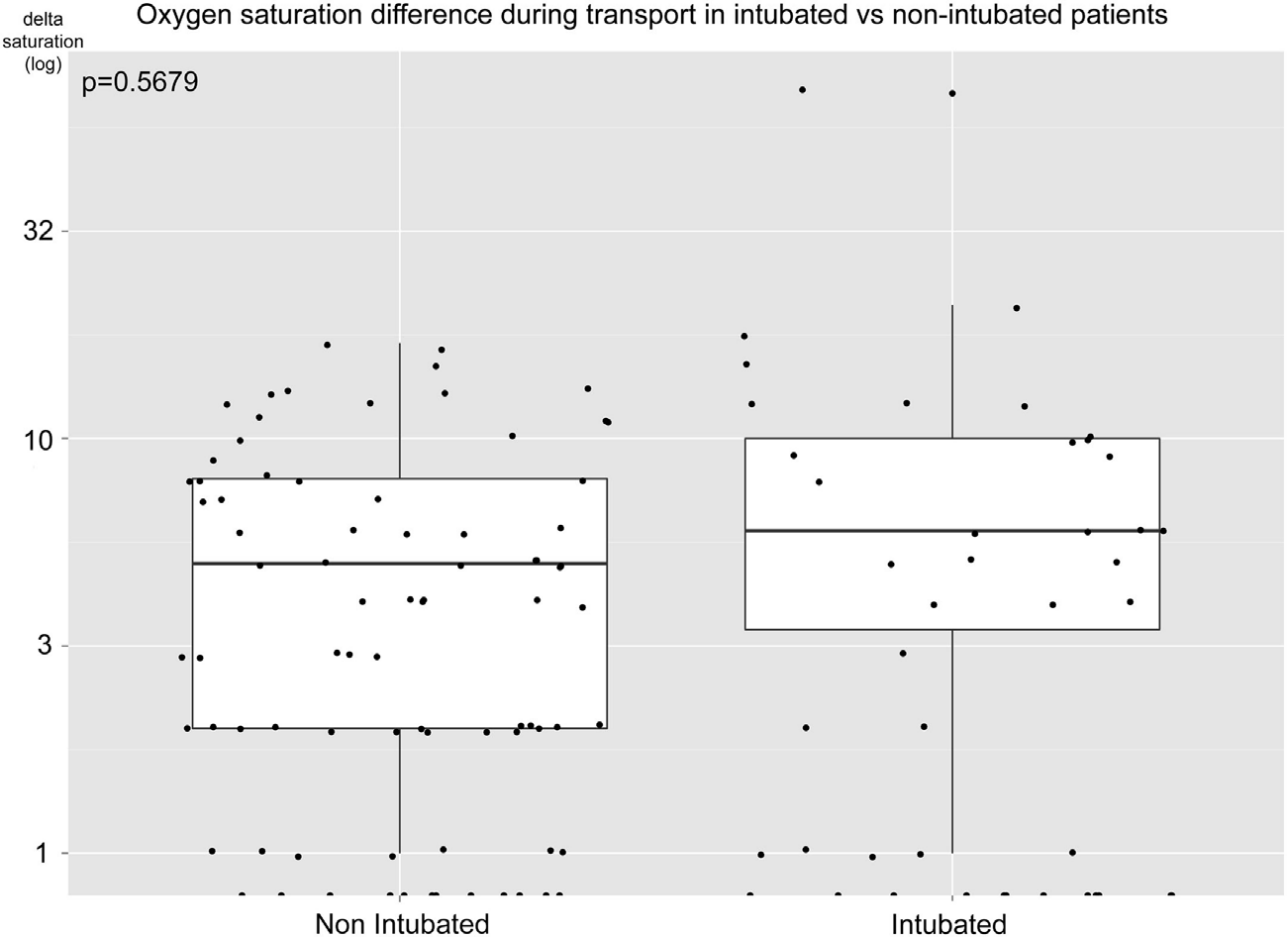
Oxygen saturation difference during prehospital transport. Difference in blood oxygen saturation (log, percentage units) for intubated and non-intubated unconscious patients during transportation from scene to hospital. Positive numbers indicate an increasing saturation. Mann–Whitney *U*-test, *p* = 0.568.

The delta-saturation during the prehospital transportation did not improve significantly (*p* = 0.568) in the intubated group (Figure 2). Thus, prehospital intubation did not significantly improve saturation on group level during transport.

In an exploratory approach, we investigated the helicopter transportations more thoroughly. Of all air transports carrying intubated patients, *n* = 18 (60%) were intubated by the EMS personnel arriving by helicopter rather than by the EMS that first arrived on scene.

## Discussion

In our modern TBI cohort from a level 1 trauma center, we found a difference between parameters correlated to prehospital intubation and functional outcome. Previously, no study has used a similar approach to analyze prehospital advance airway management in this patient group. Prehospital hypotension, pupil unresponsiveness, high energy trauma, longer distance to hospital, and helicopter transportation were independently associated with an increased intubation frequency, and among them, only pupil unresponsiveness was an independent outcome predictor. The added effect of prehospital intubation did not significantly influence outcome. Moreover, prehospital hypoxia was not associated with an unfavorable outcome in the multivariable analysis and while some patients clearly suffered from this condition, the EMS on scene did not primarily focus on this parameter when deciding on prehospital intubation. The failure to show an independent association between hypoxia and an unfavorable outcome could mean that the correct patients were intubated. This could be seen as the medical professionals making the right decision and treating the patients appropriately such that the expected effect of hypoxia (negative) is ameliorated. Further, the discrepancy between factors correlated with intubation and outcome, as well as EMS primarily not intubating because of hypoxia, could explain why this study, and the trauma literature, have failed to show a robust association between PHETI and outcome.

### Parameters Easily Assessable on the Scene Were Associated With Intubation

As suggested by the implemented guidelines (7), low level of consciousness and long distance to the hospital were factors associated with an increased rate of prehospital intubation, together with prehospital hypotension, pupil unresponsiveness, high energy trauma, and if a helicopter was used for transport. Thus, the EMS’ decision to intubate appear guided by factors involved in the field triage criteria for trauma steering (50). In the prehospital airway management literature, different guidelines apply but to intubate unconscious patients is a general rule (51). Naturally, the guidelines applied in different studies determine which parameters that would be most frequently associated with prehospital intubation. Unfortunately, many studies fail to adequately describe these and may define it as “Standard guidelines for the triage of trauma victims are used” (52). Directly analyzing which parameters that are associated with prehospital intubation has never been performed in a similar fashion in a TBI cohort. Previously, unconsciousness, respiratory insufficiency, and cardiac arrest have been described as predictors of on scene intubation in a mixed prehospital patient cohort (53), thus similar, but not identical, to our TBI cohort. Analogously to our findings, groups have seen that air transportation results in an increasing frequency of intubation (52). In our region, helicopters are often used for long distance transports from rural areas, where predominantly high-energy, motor vehicle accidents occur. We saw a marked increase in intubation frequency using helicopter transportation. In theory, the EMS in the helicopter should not be more prone to prehospital intubation than any other EMS. After thorough investigation of these cases, we believe that the addition of another EMS individual at the scene assisting in the procedure is the reason why intubation was more frequently performed in the helicopter sub-group, and not due to more severe injuries. The association between intubation and longer on-scene time is presumably not related to the severity of injury, but the extra time on-scene necessary to perform the intubation. It could also be an effect of the high frequency of helicopter use in the intubated cohort as the helicopter was often recruited after the first EMS crew had arrived on scene, thereby delaying arrival. In aggregate, it seems like the EMS intubated according to the implemented guidelines and based on parameters easily accessible on the scene.

### Surrogate Markers of Brain Injury Severity Were Associated With Outcome

Chesnut and co-workers highlighted the importance of prehospital hypoxia (and hypotension) and its role as an unfavorable outcome predictor using the Traumatic Coma Databank (2), something that has also been shown by the IMPACT study group (3) as well as other groups (54). This has resulted in airway management being a cornerstone in prehospital care of unconscious TBI patients, so as to ensure sufficient oxygen delivery to the injured brain (55, 56).

At the scene, as seen in this study, it is extremely difficult for the EMS to assess the extent and severity of the intracranial lesion and determine which patients have the most extensive, brain injury and thus who would probably be most suited for sedation and endotracheal intubation in order to prevent secondary injury development. Unexpectedly, prehospital hypotension and hypoxia were not independently associated with unfavorable outcome in our study, even if this could indicate that these conditions are properly managed. Moreover, many of the historical cohorts [used by Chesnut and IMPACT (2, 48)] did not report on the time from trauma to EMS arrival, but as these are cohorts from the 1970s to the 1990s, it is presumably longer than what was seen in our study (median 11 min). As the severity of these secondary insult depends on the time that the patient is exposed by them (8, 57), they would influence outcome to a lower extent in our cohort compared to many others.

The use of field triage criteria probably explain why the Stockholm CT score (40) and 12–48 h peak concentration of the brain enriched protein (“biomarker” of tissue fate) S100B (38), the two parameters that most strongly correlated to long-term functional outcome in the study, were not correlated to prehospital intubation. Other parameters correlated to an unfavorable outcome in the unconscious cohort were high age, pupil unresponsiveness [both strong, independent IMPACT predictors of poor outcome in TBI (48)], and increased NISS. These parameters presented similar pseudo-*R*^2^ for outcome prediction as in the IMPACT cohort (48). Age is an important aspect in this study, as increasing age was a predictor for unfavorable outcome, while a *decreasing* age was correlated to prehospital intubation (albeit not independently, presumably because of high co-variance between younger patients and the parameter high-energy trauma). Thus, it is seemingly not the patients who have the highest risk for an unfavorable outcome related to the TBI injury that are intubated on scene, although they could be expected to benefit most from an improved airway management during transport. Why NISS was superior to ISS in outcome prediction may be due to the fact that ISS is more influenced by extracranial trauma than NISS (58). As previously have been pointed out by studies investigating prehospital intubation and its effect on outcome is the fact that intubation is performed on patients with more severe injuries (28). This is something that can be seen in our study as well, as if the whole cohort of conscious patients were taken into account (Table S3 in Supplementary Material), prehospital intubation came out as a negative outcome predictor in the univariate analysis. Altogether, surrogate markers of brain injury severity were strongly associated with outcome in our study, creating a discrepancy to parameters associated with prehospital intubation.

### Intubation Frequency Increased with Distance, but Unfavorable Outcome Did Not

The EMS in Stockholm showed an intubation rate of 41% in unconscious patients and about 50% was intubated if the transport exceeded 10 km (Figure 1). While some studies recommend endotracheal intubation for longer transports (7, 59), there is no strong evidence suggesting that it improves outcome or ensures oxygen delivery. However, a longer travel distance for EMS personnel has been shown to be unfavorable for outcome in rural settings (60). The authors of that study noted that the mean distance to the scene for patients that died was 9.33 miles (15 km) compared to 7.71 miles (12.5 km) for patients that survived, thus similar distances as in our study (including similar transportation times) (60). Importantly, this study does not mention the use of helicopters, which could explain the discrepancy seen in our cohort.

Grosmann et al. has shown that if the response time is longer than 30 min, there is an increase in unfavorable outcome in trauma cohorts (61). Even though many transfers were from peripheral islands in the Stockholm archipelago (median distance between scene of accident to the nearest hospital was 11.8 km) in our cohort, the median response time was as short as 11 min. Almost all of our patients had a time duration from alarm to arrival at the hospital underneath 1 h, thus falling within the “golden hour,” a cornerstone of many trauma systems when the risk of unfavorable outcome increases (22). This could be why we did not detect any association between transportation times and outcome. Further, recent findings suggest that this time-frame may not be as important as it once was for outcome in TBI patients (24, 62), presumably as some treatment can be provided in the prehospital setting. It is a difficult compromise to decide if either stay on scene and optimize the patient versus to quickly load the patient for transportation (“scoop and run”). The EMS for the intubated cohort spend in average 12 min additional on scene as compared to non-intubated patients (14 versus 26 min); however, this was not associated with any unfavorable outcome. This is supported by a recent meta analyses showing that an extended on-scene time is not associated with an increased risk for unfavorable outcome in trauma patients (27). In summary, we could not show that increased transportation time and distance were associated with increased risk of unfavorable outcome, which could be explained by rapid transports and adequate prehospital treatment in our cohort.

### Hypoxia Was Not a Key Reason for PHETI

In contrast to other studies in the field, we had unique data as to why the EMS performed prehospital intubation. This revealed that “decreased level of consciousness” (40%), “ineffective ventilation” (18%), and “impending airway obstruction” (14%) were the most common causes and, only in 3%, was the reason purely hypoxia (Table 5). While there were patients with hypoxia at the scene (18% in the unconscious cohort), this indicates that other priorities were taken instead of the hypoxic threshold of a saturation of 90%. Because of our set-up to compare endotracheal intubation with everything else, a situation arises where supraglottic devices such as oropharyngeal, nasopharyngeal, and laryngeal mask or even bag valve masks could have been used to improve oxygenation (which would presumably be escalated to endotracheal intubation, but only if necessary) versus a cohort that had endotracheal intubation. There is evidence indicating that these methods of non-intubated advanced airway managements are equally good as endotracheal intubation when looking at survival (63, 64) is a safer way to secure the airway (65), and even shows improved outcome in non-trauma cohorts (66). Presumably, as oxygen saturation is such a common treatment goal for EMS at the scene as soon as the pulse oximetry device has been deployed, it cannot be adequately used as an outcome predictor any more. In economics, this is referred to as the Goodhart’s Law (“when a measure becomes a target, it ceases to be a good measure”) (67). This is a similar route as the intracranial pressure (ICP) metric has taken in TBI studies, as with modern therapy intensities, ICP is such a targeted metric that only mortality can be discriminated in observational studies, for patients with refractory high ICP levels (68). Luckily, there were no patients with refractory low levels of prehospital oxygen saturation following EMS arrival on scene. Moreover, as can be seen in Figure 2, there were no differences in oxygen saturation between intubated and non-intubated patients during transport form scene to the hospital. To our knowledge, delta saturation during transport has not been previously reported in this fashion. Figure 2 clearly shows that while there were two outliers in the intubated group with large improvements in saturation, the average patients improved equally well during transport independent of airway management. This is in line with the theme of this study, where the EMS seems to escalate airway management if necessary to ensure oxygen delivery. A meta-analysis from 2015 revealed that clinical experience of the EMS is a significant predictor of survival in prehospital intubated TBI patients (69). Similar findings have been reported with physicians having a greater chance of a successful prehospital intubation as compared to nurses (70), as well as less prehospital hypoxia during transport (71). However, in our study, we could not find any differences in outcome in patients intubated by nurses as compared to prehospital physicians, which could be a positive result of the training provided to the EMS following the implementation of the Scandinavian guidelines. However, as the incidence of unsuccessful intubation is low, comparison is difficult in our study.

In summary, hypoxia alone was an uncommon reason for PHETI, presumably due to a general escalated airway management difficult to assess in a retrospective setting. Our findings support that the EMS should only spend time on endotracheal intubation on scene if the patient desaturates despite other types of non-invasive airway management techniques.

### The Complexity of Analyzing Efficacy of Prehospital Interventions in a Retrospective Cohort

A great number of studies have analyzed the association between outcome and prehospital intubation in retrospective trauma cohorts, where some have shown improvement (52, 72, 73), and others deterioration and an unfavorable outcome (74–76), for the intubated cohort. We believe, as we have shown in this study that it is difficult to determine the benefit of prehospital intubation as the EMS will assess every patient individually and determine, using clinical experience, and “hidden” skills difficult to detect using these types of studies. Moreover, prehospital intubation is likely to be performed more on patients assessed to be sicker, possibly with more severe pre-morbidities. This integrated qualified and on the fly assessment is hard to quantify and may introduce a treatment bias. While we could not detect any general improvement of prehospital intubation for unconscious patients, for the individual patient, prehospital intubation may very well be an escalated therapy that is beneficial, and/or even life-saving.

A main finding and conclusion of this study is that, due to multiple confounders and possible interactions in the logistically complex prehospital situation, the merits or dangers of prehospital intubation are difficult to adequately assess in a retrospective study. As has been previously mentioned by other groups, well-designed prospective study protocols are warranted to answer this question (77, 78), but even then it will be difficult in the heterogeneous injury as TBI. In aggregate, this study suggests that decisions to intubate or not at the scene are based on judgments that are multi-factorial and hard to quantify for analysis, but are generally correct in the study region.

### Limitations

There are limitations to this study. First, the retrospective method is in itself a limitation and, in this case, a retrospective registry-based study on a single, relatively low volume, trauma center. Still, as we captured data over several years, we believe we have achieved a good sample size, which reflects the full population of patients at our trauma center and could be extrapolated to similar regions in Europe and North America. Second, some data were missing from our datasets, which we, according to standards within the field, imputed. While we retain the uncertainty of the non-imputed dataset, conclusions from heavily imputed data (such as energy level of the trauma) should be drawn with caution. Third, we were not able to control for pre-existing medical conditions or comorbidities, which of course might have influence on the results. However, as the cohort has a relatively low median age, particularly in the intubated group, fewer comorbidities are expected. Fourth, while we included different injury scores including NISS, ISS, and AIS, we did not look specifically at subcomponents of these as to highlight if thoracic injuries would be more associated with intubation. We do plan to better stratify these injuries and the importance of them in upcoming studies of our TBI population. Finally, as the trauma database we recruited our patients from is in itself a selected group of TBI patients (consisting primarily of severe and moderate TBI patients), and therefore, findings might not apply to all TBI patients in other regions with different EMS and trauma systems. Yet, this is the full population of the most severely injured TBI patients, a cohort that we think is most important to study, when analyzing prehospital airway management.

Despite these caveats, to our knowledge, this is one of the first studies to incorporate several aspects of the pre-injury management into assessment of endotracheal intubation, such as reason for intubation, saturation differences during transport, intubation frequency over distance, and intubation’s potential effect on long-term functional outcome, in both uni- and multivariable models, in a TBI cohort.

## Conclusion

Parameters associated with prehospital intubation and long-term outcome showed discrepancy in our study. This may indicate that the decisions to intubate or not at the scene, based on judgments that are multi-factorial and hard to quantify for analysis, are clinically appropriate in the study region. Difficulties with retrospective studies in an area with complicated logistics and hard to document clinical evaluations in the field become evident and can question the validity of findings. With this taken into consideration, our results support that the EMS should only spend time on PHETI if the patient desaturates despite other types of non-invasive airway management techniques. Large multi-center prospective studies with structured protocols in this area will be affected by logistic and ethical considerations.

## Ethics Statement

The study received ethical approval from the Regional Ethical Review Board in Stockholm reference numbers 2007/1113-31, 2010/1979-32, 2013/1718-32, 2014/691-32, and 2015/1675-31/1.

## Author Contributions

RW planned the study, collected data, and drafted the manuscript. B-MB planned the study and aided in drafting of the manuscript. DN planned the study, aided with statistical analyses, and drafting of the manuscript. MS planned the study and aided in the drafting of the manuscript. AH planned the study and aided in the drafting of the manuscript. ET planned the study, collected data, performed the statistical analyses, and drafted the manuscript.

## Conflict of Interest Statement

The authors declare that the research was conducted in the absence of any commercial or financial relationships that could be construed as a potential conflict of interest.

## Abbreviations

ACOS, American College of Surgeons; AIS, Abbreviated Injury Scale; CPR, cardiopulmonary resuscitation; ED, Emergency department; EMCC, Emergency Medical Communications Centre; EMS, Emergency medical services; EMT, Emergency medical technicians; GCS, Glasgow Coma Scale; GOS, Glasgow Outcome Score; ICU, intensive care unit; ISS, injury severity score; KSS, Karolinska University Hospital in Solna; LOS, length of stay (days); PHETI, prehospital endotracheal intubation; ROSC, return of spontaneous circulation; RTS, revised trauma score; RR, respiratory rate; SBP, systolic blood pressure; SCC, Stockholm County Council; TBI, traumatic brain injury.
