# Supplementary material for: Prehospital Intubation and Outcome in Traumatic Brain Injury—Assessing Intervention Efficacy in a Modern Trauma Cohort
Source: Front Neurol. 2018 Apr 10;9:194. doi: 10.3389/fneur.2018.00194 (PMC5903008; doi:10.3389/fneur.2018.00194)
Supplement: Supplementary file 1 [file Table_1.docx]

Supplementary Table 1 to “Prehospital intubation and outcome in traumatic brain injury – Assessing intervention efficacy in a modern trauma cohort.”

Supplementary Table 1 Patients demographics, the whole dataset.

| **Demographics** | Total n=458 patients | Missing |
| --- | --- | --- |
|  |  |  |
| Sex, male (%) | n=335 (73%) |  |
| Age, mean years (SD) | 47.5 (19.5) |  |
| **Trauma mechanism** |  |  |
| Mechanism (according to Utstein) |  |  |
| 1 Traffic: MVA | n=24 (5%) |  |
| 2 Traffic: Motorcycle accident | n=15 (3%) |  |
| 3 Traffic: Bicycle accident | n=20 (4%) |  |
| 4 Traffic: Pedestrian | n=24 (5%) |  |
| 5 Traffic: Other | n=11 (2%) |  |
| 6 Gunshot wound | n=6 (1%) |  |
| 7 Stab wound | n=4 (1%) |  |
| 8 Struck by blunt object | n=56 (12%) |  |
| 9 Low energy fall | n=158 (35%) |  |
| 10 High energy fall | n=137 (30%) |  |
| 12 Other | n=4 (1%) |  |
| Multitrauma, yes (%) | n=131 (29%) |  |
| High Energy trauma (according to ATLS), yes (%) | n=102 (22%) | n=249 (54%) |
| **Upon EMS arrival at scene of accident** |  |  |
| Unconscious at the scene of accident, yes (%) | n=178 (39%) |  |
| Hypotension at the scene of accident, yes (%) | n=15 (3%) | n=57 (12%) |
| Hypoxia at the scene of accident, yes (%) | n=41 (9%) | n=37 (8%) |
| Obstructive airway at the scene of accident, yes (%) | n=31 (7%) |  |
| Physician performing the intubation | n=28 (43%) |  |
| Endotracheal intubation at the scene of accident (including n=4 conscious patients intubated) | n=65 (14%) |  |
| Patients in need of several intubation attempts | n=10 (15%) |  |
| Patients deemed to need intubation but where attempts were unsuccessful (% of intubation attempts) | n=9 (12%) |  |
| Physician performing the intubation (% of intubation attempts) | n=21 (30%) |  |
| **Transportation parameters** |  |  |
| Distance from scene of accident to the hospital, median kilometers (IQR) | 11.8 (5.4-21.1) |  |
| Time from alarm until hospital arrival, hh:mm:ss (median, IQR) | 00:40:32 (00:31:20 - 00:54:12) | n=1 (0.5%) |
| Time from alarm until arrival at scene, hh:mm:ss (median, IQR) | 00:11:09 (00:07:31 - 00:17:32) | n=5 (1%) |
| On-scene time, hh:mm:ss (median, IQR) | 00:15:52 (00:11:16 - 00:21:37) | n=6 (1%) |
| Time from scene until hospital arrival, hh:mm:ss (median, IQR) | 00:10:54 (00:07:17 - 00:16:15) | n=4 (1%) |
| **Admission parameters** |  |  |
| Positive blood ethanol level at hospital admission, yes (%) | n=175 (38%) | n=31 (7%) |
| Pupil unresponsiveness at hospital admission, yes (%) | n=78 (17%) | n=9 (2%) |
| Head Abbreviated Injury Score (AIS) |  | n=7 (2%) |
| Head AIS 1 | n=0 |  |
| Head AIS 2 | n=10 (2%) |  |
| Head AIS 3 | n=93 (20%) |  |
| Head AIS 4 | n=124 (27%) |  |
| Head AIS 5 | n=224 (49%) |  |
| Injury Severity Score (ISS) (median, IQR) | 25 (16-27) | n=7 (2%) |
| New Injury Severity Score (NISS) (median, IQR) | 34 (27-50) | n=7 (2%) |
| S100B at hospital admission, median µg/L (IQR) | 2 (0.6-4.6) | n=251 (55%) |
| S100B 12-48 hours after injury, median µg/L (IQR) | 0.3 (0.2-0.6) | n=122 (27%) |
| **Admission radiology** |  |  |
| Stockholm CT-score (median, IQR) | 2 (1.5-3.0) |  |
| Rotterdam CT Classification |  |  |
| Rotterdam CT-score 1 | n=26 (6%) |  |
| Rotterdam CT-score 2 | n=60 (13%) |  |
| Rotterdam CT-score 3 | n=175 (38%) |  |
| Rotterdam CT-score 4 | n=112 (24%) |  |
| Rotterdam CT-score 5 | n=66 (14%) |  |
| Rotterdam CT-score 6 | n=19 (4%) |  |
| Marshall CT Classification |  |  |
| Marshall CT Grade I | n=3 (1%) |  |
| Marshall CT Grade II | n=150 (33%) |  |
| Marshall CT Grade III | n=39 (9%) |  |
| Marshall CT Grade IV | n=0 |  |
| Marshall CT Grade V+VI (Mass lesion) | n=266 (58%) |  |
| **Outcome parameters** |  |  |
| Hospital Length of Stay, median days (IQR) | 12 (5-26) |  |
| ICU length of stay, median days (IQR) | 4 (0.5-14) |  |
| In-hospital Mortality | n=50 (11%) |  |
| 6-12 months Glasgow Outcome Score (GOS) 1 | n=67 (15%) |  |
| GOS 2 | n=4 (1%) |  |
| GOS 3 | n=106 (23%) |  |
| GOS 4 | n=148 (32%) |  |
| GOS 5 | n=133 (29%) |  |
| Long-term favorable outcome (GOS 4-5) | n=281 (61%) |  |

Patient demographics, trauma mechanism, admission parameters and outcome for the whole cohort (n=458). MVA = Motor vehicle accident, CT = Computerized Tomography, ICU = Intensive Care Unit, SD = Standard deviation, IQR = Interquartile range.
