# Supplementary material for: Prehospital Intubation and Outcome in Traumatic Brain Injury—Assessing Intervention Efficacy in a Modern Trauma Cohort
Source: Front Neurol. 2018 Apr 10;9:194. doi: 10.3389/fneur.2018.00194 (PMC5903008; doi:10.3389/fneur.2018.00194)
Supplement: Supplementary file 2 [file Table_2.docx]

Supplementary Table 2 to “Prehospital intubation and outcome in traumatic brain injury – Assessing intervention efficacy in a modern trauma cohort.”

Supplementary Table 2. Parameters correlated to intubation using the whole dataset (n=458)

| **Univariate** | p-value | Nagelkerke’s Pseudo-R^2^ | Correlation coefficient |
| --- | --- | --- | --- |
| Parameter |  |  |  |
| Age | <0.001 | 0.063 | - |
| Gender | 0.429 | NS | NS |
| Unconscious * | <0.001 | 0.361 | + |
| Multitrauma | <0.001 | 0.136 | + |
| High/low energy trauma (if High) * | <0.001 | 0.258 | + |
| Positive blood ethanol | 0.307 | NS | NS |
| Prehospital hypoxia | 0.003 | 0.036 | + |
| Prehospital hypotension * | <0.001 | 0.086 | + |
| Pupil responsiveness | <0.001 | 0.158 | + |
| Stockholm CT Score | <0.001 | 0.052 | + |
| Head AIS | 0.103 | NS | NS |
| ISS | <0.001 | 0.178 | + |
| NISS | <0.001 | 0.104 | + |
| S100B admission | <0.001 | 0.099 | + |
| S100B 12-48 hours | 0.851 | NS | NS |
| Distance from trauma to hospital | 0.001 | 0.045 | + |
| Transportation (if by helicopter) * | <0.001 | 0.173 | + |
| Time from alarm to hospital arrival | 0.009 | 0.026 | + |
| Time for EMS to reach the trauma scene | 0.824 | NS | NS |
| Time for EMS on scene | <0.001 | 0.094 | + |
| Time from scene to hospital arrival | 0.732 | NS | NS |
| **Multivariable** |  | Adjusted pseudo-R^2^ |  |
| * Significant in multivariable model towards intubation | <0.001 | 0.521 |  |

Parameters significant in a bivariate regression analysis versus pre-hospital intubation with an un-imputated dataset, p-value for significance, Nagelkerke’s pseudo-R^2^ for the explained variance and correlation coefficient if an increase of the parameters was positively or negatively correlated to pre-hospital intubation. In the multivariate proportional odds model, an imputated dataset was used. Due to co-variance of the parameters, Time for EMS on scene was the only time duration that was used in the multivariate analysis. NS = Not significant, EMS = Emergency Medical Services, CT = Computerized tomography, AIS = Abbreviated Injury Score, ISS = Injury Severity Score, NISS = New Injury Severity Score
