# Supplementary material for: Prehospital Intubation and Outcome in Traumatic Brain Injury—Assessing Intervention Efficacy in a Modern Trauma Cohort
Source: Front Neurol. 2018 Apr 10;9:194. doi: 10.3389/fneur.2018.00194 (PMC5903008; doi:10.3389/fneur.2018.00194)
Supplement: Supplementary file 3 [file Table_3.docx]

Supplementary Table 3 to “Prehospital intubation and outcome in traumatic brain injury – Assessing intervention efficacy in a modern trauma cohort.”

Supplementary Table 3. Parameters correlated to outcome (GOS1-5) in the whole dataset (n=458).

| **Univariate** | p-value | Nagelkerke’s Pseudo-R^2^ | Correlation coefficient |
| --- | --- | --- | --- |
| Parameter |  |  |  |
| Age * | <0.001 | 0.130 | - |
| Gender | 0.541 | NS | NS |
| Unconscious | <0.001 | 0.087 | - |
| Multitrauma | 0.008 | 0.022 | + |
| High/low energy trauma (if High) | 0.031 | 0.035 | - |
| Positive EtOH | 0.002 | 0.024 | + |
| Prehospital hypoxia * | <0.001 | 0.042 | - |
| Prehospital hypotension | 0.123 | NS | NS |
| Prehospital intubation | <0.001 | 0.031 | - |
| Pupil responsiveness * | <0.001 | 0.099 | - |
| Stockholm CT Score * | <0.001 | 0.256 | - |
| Head AIS | <0.001 | 0.055 | - |
| ISS | <0.001 | 0.095 | - |
| NISS * | <0.001 | 0.144 | - |
| S100B admission | <0.001 | 0.151 | - |
| S100B 12-48 hours * | <0.001 | 0.193 | - |
| Transport (if by helicopter) | 0.142 | NS | NS |
| Distance from trauma to hospital | 0.236 | NS | NS |
| Time from alarm to hospital arrival | 0.923 | NS | NS |
| Time for EMS to reach the trauma scene | 0.967 | NS | NS |
| Time for EMS on scene | 0.375 | NS | NS |
| Time from scene to hospital arrival | 0.268 | NS | NS |
| **Multivarible** |  | Adjusted Pseduo-R^2^ |  |
| * Significant in multivariable models toward outcome | <0.001 | 0.453 |  |
| If pre-hospital intubation is added | 0.123 | 0.452 |  |

Parameters significant in a proportional odds regression analysis versus outcome (GOS1-5) with an un-imputated dataset, p-value for significance, Nagelkerke’s pseudo-R^2^ for the explained variance and correlation coefficient if an increase of the parameters was positively or negatively correlated to an increase in GOS (better outcome). In the multivariate proportional odds model, an imputated dataset was used. Due to co-variance of the parameters, S100B 12-48 hours was preferred to admission S100B, and NISS was preferred to AIS and ISS in the model. A step-up model was used to see if pre-hospital intubation added independent information to the multivariate model. NS = Not significant, EMS = Emergency Medical Services, CT = Computerized tomography, AIS = Abbreviated Injury Score, ISS = Injury Severity Score, NISS = New Injury Severity Scor
